# Retrospective analysis of postoperative adjuvant therapy and survival in T4 esophageal squamous cell carcinoma: a dual-cohort study using SEER and Chinese multicenter data

**DOI:** 10.3389/fmed.2026.1782089

**Published:** 2026-03-31

**Authors:** Zhi-Chen Xu, Jian-Cheng Li

**Affiliations:** 1 First Hospital of Quanzhou Affiliated to Fujian Medical University, Quanzhou, China; 2 Fujian Medical University Cancer Hospital, Fujian Cancer Hospital, Fuzhou, China

**Keywords:** adjuvant therapy, dual-cohort study, esophageal cancer, radiation therapy, surgery

## Abstract

**Background:**

T4N0-3M0 esophageal squamous cell carcinoma is traditionally considered unresectable, and definitive chemoradiotherapy remains the standard of care. Selected patients who achieve R0 resection may obtain superior survival, yet the value of adjuvant radiotherapy or chemotherapy in this setting remains undefined.

**Methods:**

We performed a retrospective dual-cohort analysis using the SEER registry (*n* = 183) and a two-center Chinese database (*n* = 54) to evaluate the survival impact of postoperative radiotherapy and chemotherapy in patients with resected T4N0-3M0 esophageal squamous cell carcinoma. Propensity-score matching, multivariable Cox regression, and restricted cubic spline analysis were used to assess survival outcomes and explore potential dose–response relationships. All Chinese patients received postoperative radiotherapy.

**Results:**

Radiotherapy (preoperative or postoperative) was independently associated with improved survival. Compared with no radiotherapy, Postoperative radiotherapy was associated with a 56% lower risk of mortality (HR 0.44, 95% CI 0.26–0.76, *p* = 0.003), while preoperative radiotherapy was associated with a 78% lower risk of mortality (HR 0.22, 95% CI 0.11–0.46, *p* < 0.001). Findings remained consistent after propensity-score matching. The survival benefit was most pronounced in node-negative patients. Postoperative chemotherapy showed no significant survival advantage on multivariable analysis. Age ≥60 and lymphovascular invasion were independent poor prognostic factors in the Chinese postoperative radiotherapy cohort.

**Conclusion:**

This study generates the hypothesis that postoperative radiotherapy may be associated with improved survival in selected T4N0-3M0 ESCC patients. These observational findings cannot establish causality. Prospective randomized trials are warranted before clinical adoption.

## 1 Introduction

Esophageal squamous cell carcinoma (ESCC) remains one of the most aggressive gastrointestinal malignancies worldwide (1). Despite recent advances in surgical techniques and peri-operative care (2, 3), outcomes for patients with locally advanced disease remain poor. Patients with T4N0-3M0 disease, characterized by full-thickness oesophageal-wall invasion and infiltration of adjacent organs, represent the greatest therapeutic challenge (4). Historically, T4 ESCC was considered unresectable, and definitive chemoradiotherapy has been the standard of care (5–7). More recent evidence indicates that highly selected patients who undergo R0 resection achieve superior overall survival compared with those treated with chemoradiotherapy alone (4, 8, 9). Consequently, an increasing number of centers now adopt a surgery-first strategy for potentially resectable T4N0-3M0 ESCC, supplemented by preoperative or postoperative radiotherapy and/or chemotherapy (4, 10, 11).

The optimal adjuvant or neoadjuvant regimen remains undefined for patients with surgically treated ESCC who have not received neoadjuvant radiotherapy. National Comprehensive Cancer Network (NCCN) guidelines (12) recommend active surveillance after R0 resection, whereas the Chinese Society of Clinical Oncology (CSCO) (13) suggests enrolment in clinical trials; both recommendations are supported by low-level evidence. Owing to the rarity of this population, prospective clinical research has been difficult. Whether additional radiotherapy or chemotherapy improves survival in patients who have undergone complete resection of T4N0-3M0 ESCC therefore remains an urgent, unanswered question.

To address this knowledge gap, we conducted a retrospective dual-cohort analysis using the Surveillance, Epidemiology, and End Results (SEER) registry (*n* = 183) and a two-center Chinese database (*n* = 54). The aims were: (1) to describe real-world use of postoperative radiotherapy and chemotherapy in patients with surgically treated T4N0-3M0 ESCC; (2) to identify independent prognostic factors and evaluate the respective roles of radiotherapy and chemotherapy; and (3) to determine the optimal radiotherapy dose. The findings may inform future guideline updates and provide a benchmark for prospective randomized trials.

## 2 Materials and methods

### 2.1 Patients

**Figure 1:**
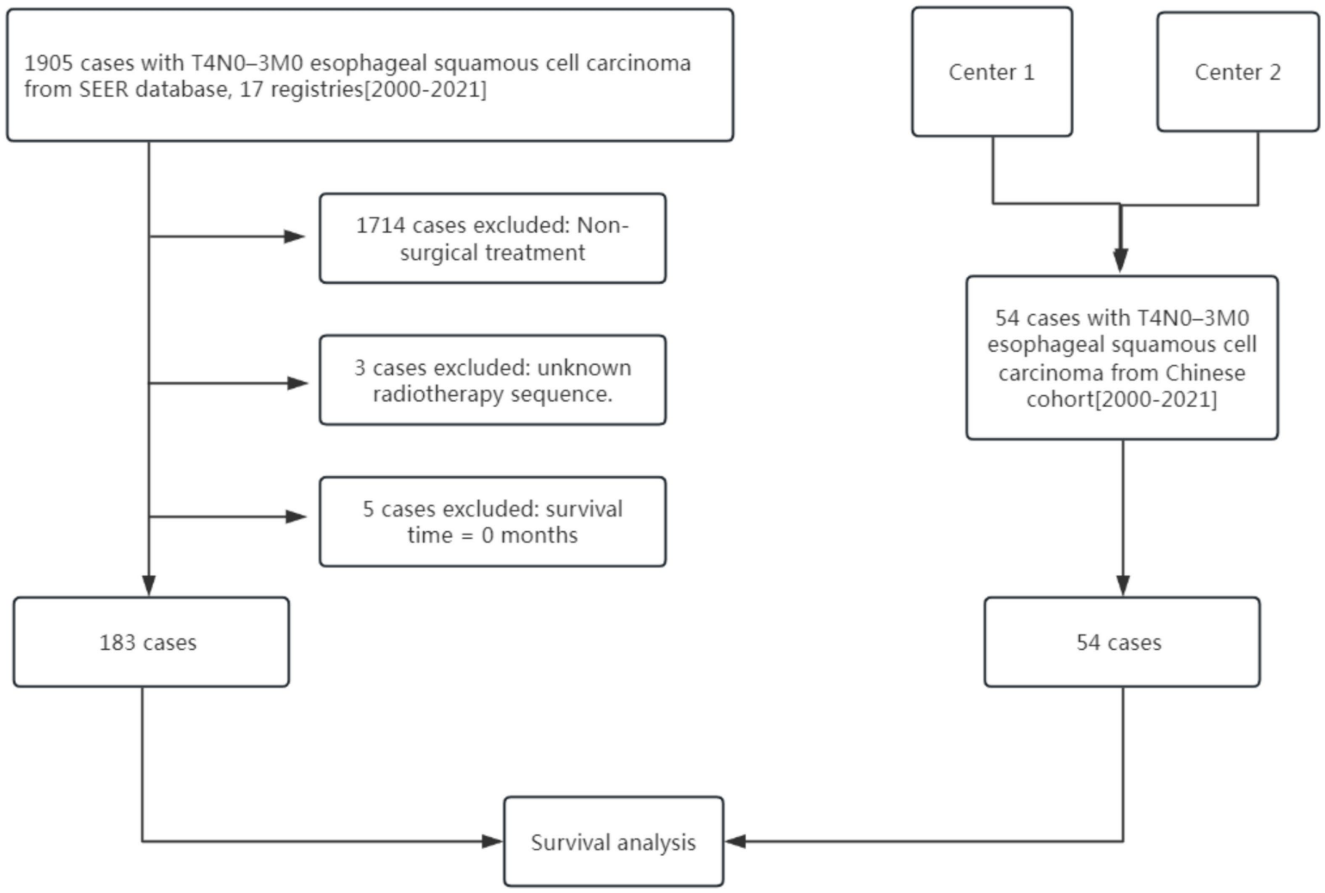
Flow chart of patient recruitment pathway.

Clinical data from 183 patients with surgically treated T4N0-3M0 oesophageal squamous-cell carcinoma identified in the SEER registry, together with 54 corresponding patients from a two-center Chinese cohort, were retrospectively analyzed. The study was approved by the Ethics Committee of the First Affiliated Hospital of Quanzhou, Fujian Medical University. Figure 1 outlines the patient-selection flow. The inclusion criteria were as follows: (1) patients with esophageal squamous carcinoma (ICD-03, 8,052, 8,053, 8,070–8,078, 8,083–8,086, 8,560); (2) patients diagnosed between 2000 and 2021; (3) Patients were staged as T4N0-3M0; (4) All patients underwent radical R0 resection. The exclusion criteria were as follows: (1) Patients had not undergone radical surgical resection; (2) Patients with missing or incomplete data on key variables, including unknown radiotherapy sequence; (3) Perioperative mortality as death within 30 days of surgery or any record with “survival months = 0” in SEER.

### 2.2 Data collection

From the SEER database we extracted demographic and clinical variables: age, sex, race, primary-site ICD-O-3 code, grade, TNM stage, surgical procedure, radiotherapy, chemotherapy and marital status at diagnosis. Outcomes were survival months, vital status and cause-specific death. Stage was assigned preferentially with AJCC 7th-edition TNM (2010–2015); if unavailable, EOD 2018 TNM (2018+) was used, followed by SEER Combined TNM (2016–2017) when both prior sources were missing. Radiotherapy categories were derived from the SEER variable “RX Summ--Surg/Rad Seq” and classified as follows: no radiation (“No radiation”), postoperative radiation (“Radiation after surgery”), or pre-operative radiation (“Radiation prior to surgery”).

All Chinese patients were restaged using postoperative pathological criteria and received postoperative intensity-modulated radiotherapy (IMRT). For this cohort we recorded the total radiotherapy dose, technique, chemotherapy regimen and number of cycles. All 54 patients underwent three-field oesophagectomy with at least 12 lymph nodes removed. Pathological examination confirmed T4 disease: all patients were classified as T4a (potentially resectable) with tumor invasion limited to the pleura, pericardium, diaphragm or peritoneum. R0 resection was achieved in every case. The postoperative clinical target volume (CTV) routinely encompassed the bilateral supraclavicular fossae, upper and middle mediastinal nodal stations, and the esophageal tumor bed. For tumors of the lower thoracic esophagus, abdominal nodal regions—including the celiac axis (No. 9), common hepatic artery (No. 8), para-aortic (No. 16a1/16a2), and retropancreatic (No. 13) nodes—were selectively contoured. The prescribed postoperative radiotherapy dose ranged from 5,000 to 6,600 cGy.

### 2.3 Statistical analysis

We used restricted cubic spline (RCS) Cox regression to explore possible non-linear relationships between radiotherapy dose (continuous) and mortality; the variable was later categorized for tabular presentation. Categorical data are summarized as counts (%). Baseline characteristics were compared with the χ^2^ or Fisher’s exact test. Survival curves were generated by the Kaplan–Meier method and compared with the log-rank test. All variables from univariate analysis were entered into a multivariable Cox model regardless of *p* value. Hazard ratios (HRs) and 95% confidence intervals (CIs) were estimated with Cox proportional-hazards models. Propensity-score matching was used to minimize confounding by indication. Dose-specific analyses employed RCS, propensity-score-matched survival, and multivariable-adjusted survival methods. *p* < 0.05 was considered significant. Analyses were performed in R (version 4.2.2; R Foundation).

## 3 Results

### 3.1 Patient characteristics

Table 1 summarizes baseline characteristics. The Chinese cohort (*n* = 54) was significantly younger: 59.3% were aged <60 years versus 36.1% in the SEER cohort (*n* = 183), and a higher proportion were men (83.3% vs. 66.7%). Most Chinese patients were diagnosed in 2010–2021 (87.0% vs. 45.4%); all were Asian and married, compared with 67.2% White and 20.2% Black patients in SEER, of whom 50.3% were married.

**Table 1:** Patient demographics and baseline characteristics.

Characteristic	Cohort		SMD (95% CI)	*p*-value
	SEER (*N* = 183)	China (*N* = 54)		
Age, *n* (%)			0.48 (0.17, 0.78)	0.002
<60	66 (36.1%)	32 (59.3%)		
≥60	117 (63.9%)	22 (40.7%)		
Sex, *n* (%)			0.39 (0.09, 0.70)	0.018
Female	61 (33.3%)	9 (16.7%)		
Male	122 (66.7%)	45 (83.3%)		
Year of diagnosis, *n* (%)			0.98 (0.67, 1.30)	<0.001
2000–2009	100 (54.6%)	7 (13.0%)		
2010–2021	83 (45.4%)	47 (87.0%)		
Race, *n* (%)			3.73 (3.28, 4.18)	<0.001
Black	37 (20.2%)	0 (0.0%)		
Other	23 (12.6%)	54 (100.0%)		
White	123 (67.2%)	0 (0.0%)		
Primary site, *n* (%)			1.15 (0.83, 1.47)	<0.001
C15.0-Cervical esophagus	33 (18.0%)	0 (0.0%)		
C15.1-Thoracic esophagus	10 (5.5%)	0 (0.0%)		
C15.2-Abdominal esophagus	1 (0.5%)	0 (0.0%)		
C15.3-Upper third of esophagus	25 (13.7%)	20 (37.0%)		
C15.4-Middle third of esophagus	47 (25.7%)	24 (44.4%)		
C15.5-Lower third of esophagus	46 (25.1%)	9 (16.7%)		
C15.8-Overlapping lesion of esophagus	7 (3.8%)	1 (1.9%)		
C15.9-Esophagus, NOS	14 (7.7%)	0 (0.0%)		
Grade, *n* (%)			1.26 (0.93, 1.58)	<0.001
1	14 (7.7%)	10 (18.5%)		
2	76 (41.5%)	42 (77.8%)		
3	68 (37.2%)	2 (3.7%)		
4	2 (1.1%)	0 (0.0%)		
X	23 (12.6%)	0 (0.0%)		
N. stage, *n* (%)			0.40 (0.09, 0.70)	0.110
0	86 (47.0%)	23 (42.6%)		
1	83 (45.4%)	21 (38.9%)		
2	8 (4.4%)	6 (11.1%)		
3	4 (2.2%)	4 (7.4%)		
X	2 (1.1%)	0 (0.0%)		
Number of lymph nodes dissected, *n* (%)			1.10 (0.78, 1.42)	<0.001
1 to 3 regional lymph nodes removed	19 (10.4%)	0 (0.0%)		
4 or more regional lymph nodes removed	114 (62.3%)	54 (100.0%)		
No/unknown	50 (27.3%)	0 (0.0%)		
Marital status at diagnosis, *n* (%)			1.41 (1.08, 1.74)	<0.001
Divorced	18 (9.8%)	0 (0.0%)		
Married	92 (50.3%)	54 (100.0%)		
Separated	2 (1.1%)	0 (0.0%)		
Single	44 (24.0%)	0 (0.0%)		
Unknown	5 (2.7%)	0 (0.0%)		
Unmarried or domestic partner	1 (0.5%)	0 (0.0%)		
Widowed	21 (11.5%)	0 (0.0%)		
Radiotherapy, *n* (%)			2.48 (2.10, 2.86)	<0.001
No radiation	63 (34.4%)	0 (0.0%)		
Radiation after surgery	45 (24.6%)	53 (100.0%)		
Radiation prior to surgery	75 (41.0%)	0 (0.0%)		
Chemotherapy, *n* (%)			3.25 (2.83, 3.67)	<0.001
Sequence unknown	30 (16.4%)	0 (0.0%)		
Chemotherapy after surgery	30 (16.4%)	46 (85.2%)		
Chemotherapy before surgery	58 (31.7%)	0 (0.0%)		
No/unknown	65 (35.5%)	8 (14.8%)		

Tumors were more often located in the upper (37.0%) or middle (44.4%) third of the oesophagus in the Chinese cohort, whereas the SEER cohort showed a higher proportion in the middle (25.7%) and lower (25.1%) thoracic oesophagus. Histologically, 96.3% of Chinese cases were well–moderately differentiated (grades 1–2) versus 49.2% in SEER. N-stage distribution did not differ significantly between cohorts (*p* = 0.110).

Lymph-node dissection was more thorough in the Chinese cohort: 100% had ≥4 nodes removed compared with 62.3% in SEER. Radiotherapy and chemotherapy patterns also differed: in SEER, 41.0% received pre-operative radiotherapy, 24.6% postoperative radiotherapy, and 64.5% chemotherapy; in contrast, all Chinese patients received postoperative radiotherapy and 85.2% received postoperative chemotherapy.

### 3.2 Survival analysis

Among the 183 SEER patients, median overall survival (OS) was 1.2 years (95% CI 0.9–1.7); 1-, 3- and 5-year OS rates were 53.2% (95% CI 46.4–61.0%), 30.7% (95% CI 24.5–38.4%) and 26.0% (95% CI 20.1–33.6%), respectively. By nodal status, the node-negative subgroup (*n* = 86) achieved a median OS of 1.1 years (95% CI 0.8–1.9) and a 5-year OS of 24.9% (95% CI 17.0–36.3%), whereas the node-positive subgroup (*n* = 95) recorded a median OS of 1.2 years (95% CI 0.9–2.0) and a 5-year OS of 26.5% (95% CI 18.5–37.8%).

Radiotherapy was associated with substantial survival prolongation. Patients who received no radiotherapy (*n* = 63) had a median OS of 0.5 years (95% CI 0.3–1.2) and a 5-year OS of 14.3% (95% CI 7.6–26.8%). Those treated with pre-operative radiotherapy (*n* = 45) achieved a median OS of 1.9 years (95% CI 1.2–4.8) and 1-, 3- and 5-year survival rates of 67.6% (95% CI 57.8–79.2%), 41.2% (95% CI 31.1–54.6%) and 36.3% (95% CI 26.4–49.8%), respectively. Patients receiving postoperative radiotherapy (*n* = 75) experienced a median OS of 1.4 years (95% CI 0.8–2.9) and 1-, 3- and 5-year survival rates of 52.6% (95% CI 39.8–69.6%), 30.7% (95% CI 19.5–48.4%) and 25.4% (95% CI 15.0–43.0%), respectively.

**Figure 2:**
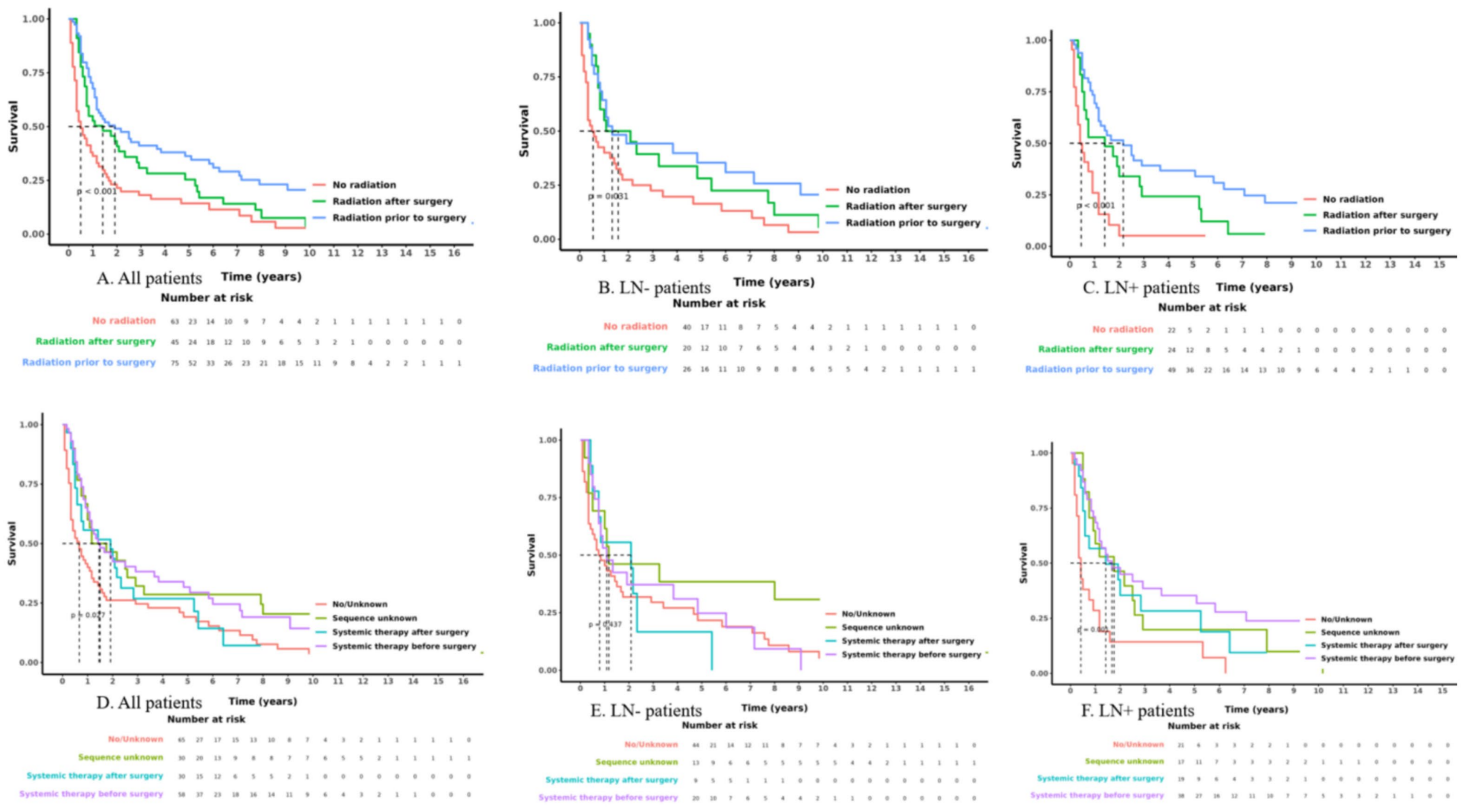
Survival curves of radiotherapy and chemotherapy in surgically treated patients with T4 esophageal squamous cell carcinoma. Panels **A–C**: Radiotherapy survival analyses for **(A)** all patients, **(B)** lymph node-negative patients, and **(C)** lymph node-positive patients (no radiotherapy, preoperative, postoperative). Panels **D–F**: Chemotherapy survival analyses for **(D)** all patients, **(E)** lymph node-negative patients, and **(F)** lymph node-positive patients (no chemotherapy, preoperative, postoperative, sequence-unknown). LN+: lymph-node–positive; LN−: lymph-node–negative.

Among the 65 patients who did not receive chemotherapy, median survival was 0.7 years (95% CI 0.3–1.2), with 1-, 3- and 5-year survival rates of 40.0% (95% CI 29.7–53.9%), 24.6% (95% CI 16.1–37.7%) and 19.1% (95% CI 11.5–32.0%), respectively. In the 118 patients who received chemotherapy, median survival was 1.5 years (95% CI 1.1–2.5), and 1-, 3- and 5-year survival rates were 60.4% (95% CI 52.2–70.0%), 33.8% (95% CI 25.9–44.0%) and 29.5% (95% CI 22.0–39.7%), respectively. Figure 2 illustrates the survival curves for radiotherapy and chemotherapy in surgically treated patients with T4 oesophageal squamous-cell carcinoma.

### 3.3 Univariate and multivariate cox regression analysis

Table 2 summarizes the univariate and multivariate survival analyses (SEER database). On univariate analysis, Both pre-operative chemotherapy (HR 0.59, 95% CI 0.40–0.86, *p* = 0.007) and postoperative radiotherapy (HR 0.63, 95% CI 0.42–0.95, *p* = 0.03) were significantly associated with reduced mortality, while chemotherapy overall showed a protective trend.

**Table 2:** Univariate and multivariate analysis of influencing factors (Cox regression).

Characteristic	Univariable			Multivariable		
	HR	95% CI	*p*-value	HR	95% CI	*p*-value
Age						
<60	–	–		–	–	
≥60	1.19	0.86, 1.67	0.297	1.17	0.79, 1.73	0.429
Sex						
Female	–	–		–	–	
Male	0.95	0.68, 1.32	0.746	1.42	0.94, 2.15	0.093
Year of diagnosis						
2000–2009	–	–		–	–	
2010–2021	0.80	0.57, 1.11	0.180	0.90	0.59, 1.37	0.621
Race						
Black	–	–		–	–	
Other	0.53	0.28, 0.99	0.048	0.53	0.26, 1.10	0.086
White	0.98	0.66, 1.45	0.918	0.96	0.59, 1.55	0.857
Primary site						
C15.0-Cervical esophagus	–	–		–	–	
C15.1-Thoracic esophagus	0.72	0.33, 1.59	0.423	0.89	0.37, 2.17	0.802
C15.2/C15.5-Abdominal esophagus and lower third of esophagus	0.76	0.47, 1.23	0.259	0.91	0.50, 1.65	0.744
C15.3-Upper third of esophagus	0.75	0.42, 1.33	0.325	0.75	0.41, 1.39	0.367
C15.4-Middle third of esophagus	0.85	0.52, 1.37	0.497	1.17	0.65, 2.09	0.597
C15.8-Overlapping lesion of esophagus	1.27	0.56, 2.90	0.565	1.28	0.50, 3.30	0.610
C15.9-Esophagus, NOS	1.10	0.57, 2.13	0.773	0.96	0.44, 2.09	0.919
Grade						
G1	–	–		–	–	
G2	1.51	0.79, 2.86	0.212	2.23	1.10, 4.52	0.027
G3	1.61	0.84, 3.07	0.148	3.07	1.49, 6.31	0.002
G4	2.43	0.54, 11.00	0.250	3.28	0.54, 19.73	0.195
GX	1.04	0.49, 2.21	0.919	2.16	0.90, 5.21	0.086
N. stage						
N0	–	–		–	–	
N1	1.00	0.72, 1.39	0.989	1.33	0.87, 2.04	0.182
N2	0.83	0.36, 1.90	0.657	1.07	0.40, 2.84	0.893
N3	0.49	0.12, 2.00	0.319	0.26	0.05, 1.30	0.101
NX	0.61	0.08, 4.40	0.624	0.22	0.03, 1.84	0.162
Number of lymph nodes dissected						
1 to 3 regional lymph nodes removed	–	–		–	–	
4 or more regional lymph nodes removed	0.97	0.57, 1.66	0.914	0.95	0.51, 1.77	0.866
No/unknown	1.38	0.78, 2.45	0.264	1.38	0.68, 2.81	0.370
Radiotherapy						
No radiation	–	–		–	–	
Radiation after surgery	0.63	0.42, 0.95	0.029	0.44	0.26, 0.76	0.003
Radiation prior to surgery	0.45	0.31, 0.66	<0.001	0.22	0.11, 0.46	<0.001
Chemotherapy						
No/unknown	–	–		–	–	
Sequence unknown	0.59	0.37, 0.94	0.025	1.51	0.72, 3.15	0.271
Chemotherapy after surgery	0.74	0.46, 1.21	0.229	1.19	0.61, 2.33	0.610
Chemotherapy before surgery	0.59	0.40, 0.86	0.007	1.63	0.79, 3.36	0.184
Marital status at diagnosis						
Divorced	–	–		–	–	
Married	0.82	0.49, 1.38	0.462	1.22	0.67, 2.22	0.515
Separated	0.46	0.11, 1.99	0.298	0.61	0.11, 3.21	0.555
Single	0.95	0.54, 1.68	0.872	1.42	0.71, 2.85	0.319
Unknown	1.18	0.35, 4.03	0.791	2.91	0.74, 11.40	0.125
Unmarried or domestic partner	0.00	0.00, Inf	0.994	0.00	0.00, Inf	0.995
Widowed	0.87	0.45, 1.69	0.681	1.21	0.56, 2.60	0.622

CI, Confidence Interval; HR, Hazard Ratio.

To minimize selection bias, all variables were entered into the multivariate cox regression simultaneously without pre-screening. Multivariate Cox regression confirmed that higher tumor grade (G2: HR 2.23, 95% CI 1.10–4.52, *p* = 0.03; G3: HR 3.07, 95% CI 1.49–6.31, *p* = 0.002) were independent adverse prognostic factors. Radiotherapy remained significantly beneficial: postoperative radiotherapy was associated with a 56% lower risk of death (HR 0.44, 95% CI 0.26–0.76, *p* = 0.003), and pre-operative radiotherapy with a 78% lower risk of death (HR 0.22, 95% CI 0.11–0.46, *p* < 0.001). In contrast, chemotherapy showed no significant effect regardless of timing.

To minimize confounding we performed three independent 1:1 propensity-score-matched analyses; all comparisons achieved covariate balance (*p* > 0.05, standardized mean differences (SMD) < 0.1) unless stated otherwise. Postoperative radiotherapy versus no radiotherapy (age, sex, race, primary site, grade, TNM stage, chemotherapy, marital status) yielded 45 matched pairs; no radiotherapy versus pre-operative radiotherapy (age, sex, race, grade, T-stage, M-stage) yielded 63 pairs; and pre-operative versus postoperative radiotherapy (age, sex, race, grade, TNM stage) yielded 45 pairs.

**Figure 3:**
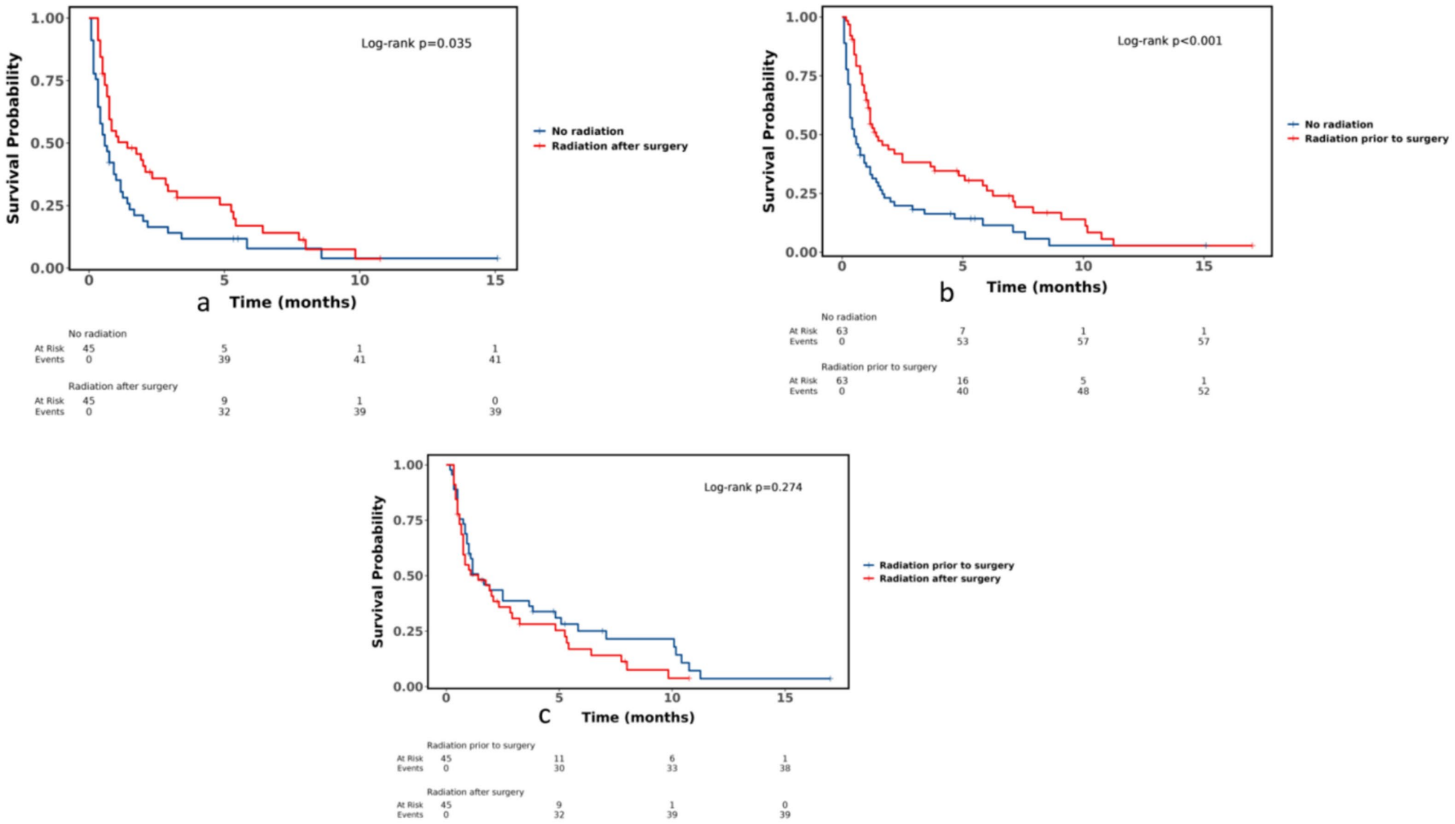
Survival curves after propensity-score-matched analysis: **(a)** no radiotherapy vs. postoperative radiotherapy; **(b)** no radiotherapy vs. preoperative radiotherapy; **(c)** preoperative radiotherapy vs. postoperative radiotherapy. Post-1: 1PSM: postoperative radiotherapy vs. no radiotherapy (age, sex, race, primary-site, grade, TNM stage, chemotherapy, and marital status) *p* > 0.05, balanced 45:45; preoperative radiotherapy vs. no radiotherapy (age, sex, race, grade, Tstage, M stage) *p* > 0.05, balanced 63:63; preoperative radiotherapy vs. postoperative radiotherapy (age, sex, race, grade, TNM stage) *p* > 0.05, balanced 45:45.

After matching, postoperative radiotherapy remained significantly associated with lower mortality compared with no radiotherapy (HR 0.62, 95% CI 0.40–0.97, *p* = 0.036), whereas pre-operative radiotherapy versus no radiotherapy yielded HR 0.53 (95% CI 0.36–0.77, *p* = 0.001). When pre-operative radiotherapy was used as the reference, postoperative radiotherapy showed a non-significant trend toward higher mortality (HR 1.28, 95% CI 0.81–2.02, *p* = 0.283). Survival curves after matching are shown in Figure 3.

In the Chinese cohort (*n* = 54), multivariate Cox regression identified age ≥60 years (adjusted HR 4.61, 95% CI 1.17–18.19, *p* = 0.029) and lymphovascular invasion (adjusted HR 21.92, 95% CI 4.44–108.16, *p* < 0.001) as independent predictors of increased mortality. After pooling both cohorts, radiotherapy remained independently associated with improved survival relative to no radiation: postoperative radiotherapy was associated with a 54% lower risk of mortality (HR 0.46, 95% CI 0.27–0.79, *p* = 0.005) and pre-operative radiotherapy with a 76% lower risk of mortality (HR 0.24, 95% CI 0.12–0.48, *p* < 0.001).

### 3.4 Subgroup analysis of radiotherapy in surgical patients with T4N0-3M0 ESCC

When stratified by nodal status, the survival benefit of radiotherapy remained most pronounced in the node-negative subset (*n* = 86). Compared with the no-radiotherapy group (*n* = 40), postoperative radiotherapy (*n* = 20) was associated with an 81% lower risk of death (HR 0.19, 95% CI 0.08–0.46, *p* < 0.001), and pre-operative radiotherapy (*n* = 26) with a 93% lower risk of death (HR 0.07, 95% CI 0.02–0.27, *p* < 0.001). Conversely, none of the chemotherapy timings conferred a significant survival advantage in this subgroup; some were even associated with higher mortality (sequence-unknown HR 5.15, 95% CI 1.47–18.02, *p* = 0.010; postoperative chemotherapy HR 2.10, 95% CI 0.72–6.11, *p* = 0.174; pre-operative chemotherapy HR 6.21, 95% CI 1.88–20.51, *p* = 0.003) (Figures 2B,E).

In the node-positive subset (*n* = 95) radiotherapy retained a protective effect: pre-operative radiotherapy remained significant (HR 0.22, 95% CI 0.08–0.60, *p* = 0.003), while postoperative radiotherapy was of borderline significance (HR 0.36, 95% CI 0.12–1.04, *p* = 0.058). Univariate analysis in this subgroup suggested a benefit for chemotherapy (sequence-unknown HR 0.42, 95% CI 0.21–0.82, *p* = 0.012; postoperative HR 0.45, 95% CI 0.23–0.90, *p* = 0.024; pre-operative HR 0.32, 95% CI 0.18–0.57, *p* < 0.001); however, these associations were no longer significant after multivariable adjustment.

Across age strata, radiotherapy remained independently protective. Among patients aged ≥60 years, postoperative radiotherapy was associated with a 67% lower risk of mortality (HR 0.33, 95% CI 0.15–0.72, *p* = 0.005) and pre-operative radiotherapy with an 86% lower risk of mortality (HR 0.14, 95% CI 0.06–0.37, *p* < 0.001). An even stronger effect was observed in those <60 years: postoperative radiotherapy HR 0.33 (95% CI 0.18–0.59, *p* < 0.001) and pre-operative radiotherapy HR 0.07 (95% CI 0.04–0.12, *p* < 0.001). For tumors located in the thoracic oesophagus, postoperative radiotherapy was associated with a 71% lower risk of mortality (HR 0.29, 95% CI 0.13–0.65, *p* = 0.002) and pre-operative radiotherapy with an 81% lower risk of mortality (HR 0.19, 95% CI 0.08–0.45, *p* < 0.001) (Figures 2C,F).

### 3.5 Analysis of postoperative radiotherapy dose

**Figure 4:**
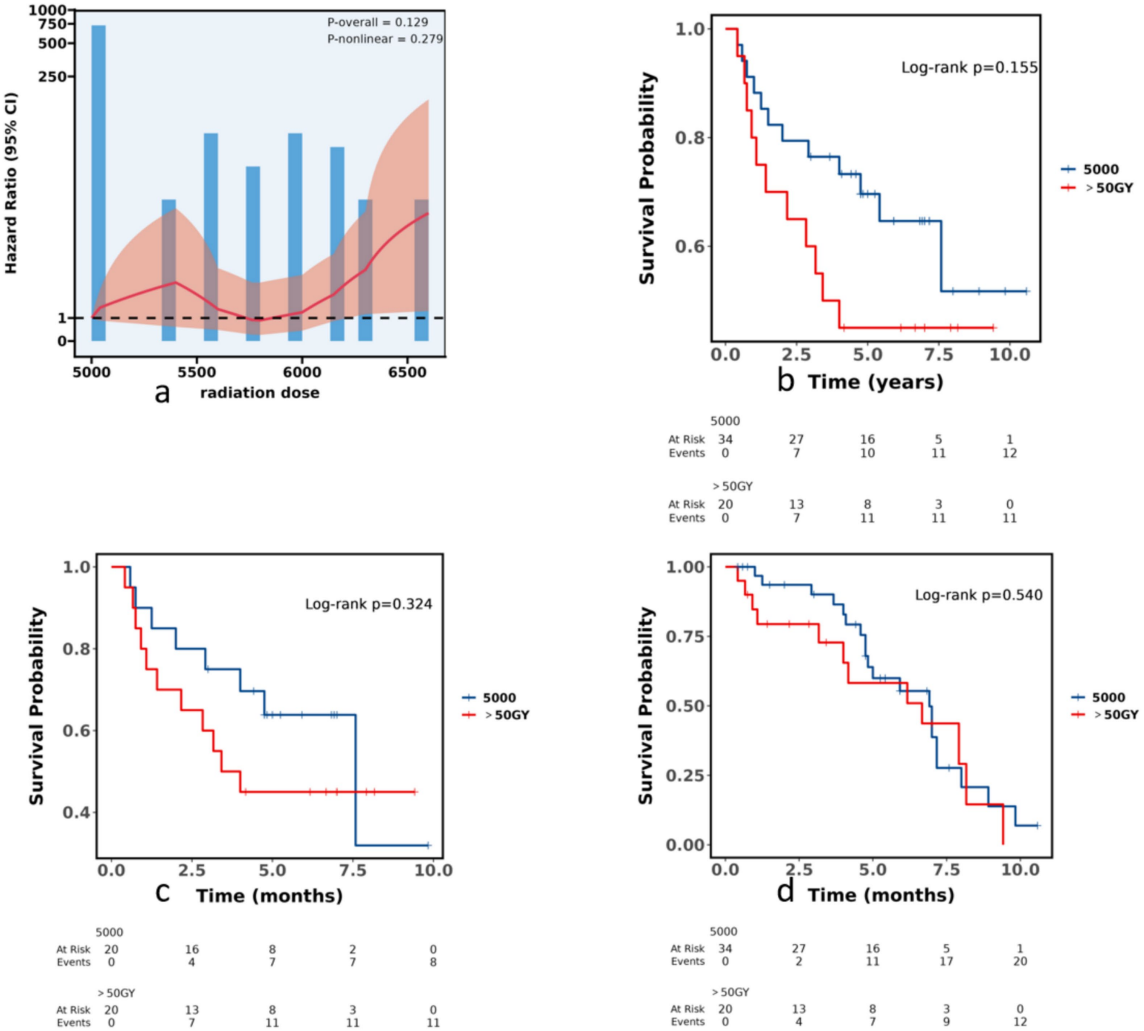
Analysis of postoperative radiotherapy dose. **(a)** Restricted cubic spline (RCS) plot of the association between radiotherapy dose and mortality. Seven knots were placed at the 2nd, 18th, 34th, 50th, 66th, 82nd, and 98th percentiles; the reference dose was 50 Gy (HR = 1). The model was adjusted for age, sex, primary site, grade, N stage and chemotherapy. P-non-linear = 0.279. **(b)** Pre-matching; **(c)** post-matching (1:1 PSM with chemotherapy as covariate, SMD < 0.1); and **(d)** multivariable-adjusted survival curves comparing >50 Gy with 50 Gy. Sample sizes: pre-PSM 34 vs. 20; post-PSM 20 vs. 20.

To investigate the potential nonlinear relationship between radiation dose and mortality risk, we applied RCS to the Chinese cohort. Figure 4a displays the RCS plot for radiotherapy dose. After fitting models with 3–7 knots, the AIC values were:170.121, 169.976, 169.884, 169.834, 169.794. The lowest AIC (169.794) corresponds to 7 knots (2nd, 18th, 34th, 50th, 66th, 82nd and 98th percentiles), which was therefore selected automatically. The RCS plot shows how the hazard ratio (HR) changes across the dose range. The risk of death remained relatively stable at doses between 50Gy and 60Gy, but rose sharply once the dose exceeded 60Gy, reaching a peak at 65Gy. Among the 54 patients, the distribution of prescribed radiotherapy doses was heavily left-skewed. The 2nd, 18th, 34th and 50th percentiles all corresponded to 5,000 cGy, indicating that half of the cohort received this dose or less. Doses then rose gradually: the 66th percentile was 5,040 cGy, while the 82nd and 98th percentiles reached 5,778 cGy and 6,291 cGy, respectively. We therefore selected 5,000 cGy as the reference cut-point. Overall, however, the association was not statistically significant (P-overall = 0.129), and there was little evidence of non-linearity (P-non-linear = 0.279). Thus, although the spline reveals some fluctuation in risk with increasing dose, these variations do not achieve conventional levels of statistical certainty.

Of the 54 patients, 62.96% (*n* = 34) received postoperative radiotherapy at 50 Gy, whereas 37.04% (*n* = 20) received >50 Gy (median 60 Gy). In the full analysis set, patients receiving >50 Gy (median 60 Gy) showed consistently lower survival probabilities than those receiving 50 Gy (median 50 Gy) across all analytical scenarios: pre-matching HR 1.80 (95% CI 0.79–4.08, *p* = 0.161), post-matching HR 1.58 (95% CI 0.63–3.93, *p* = 0.329), and multivariable-adjusted HR 1.24 (95% CI 0.60–2.57, *p* = 0.561). Before propensity-score matching, the radiotherapy dose distribution was 50 Gy vs. >50 Gy (*n* = 34 vs. *n* = 20). After 1:1 nearest-neighbor matching (with chemotherapy as a covariate), SMD<0.1, yielding a balanced cohort of 50 Gy vs. >50 Gy (*n* = 20 vs. *n* = 20). Figures 4b–d display pre-matching, post-matching, and multivariable-adjusted (age, sex, primary site, grade, N stage, chemotherapy) survival curves for radiotherapy dose >50 Gy versus 50 Gy.

## 4 Discussion

T4N0-3M0 ESCC is still widely regarded as “unresectable” and is routinely referred for definitive chemoradiotherapy, yet long-term outcomes with this strategy remain disappointing (14). Accumulating retrospective series (4, 15–20) now show that, after rigorous patient selection, R0 resection can outperform non-surgical management, but whether any adjuvant therapy is required when upfront surgery is performed without neoadjuvant treatment remains unresolved. In the absence of prospective randomized data, the NCCN guideline recommends active surveillance, and the CSCO guideline advises enrolment in clinical trials only.

The present study was restricted to surgically resectable T4 disease—specifically T4a; all patients underwent R0 resection. Our findings should not, therefore, be extrapolated to T4b tumors, for which trimodality therapy remains controversial and primary surgery is generally contraindicated. By pooling 183 R0-resected T4a cases from SEER with 54 cases from two Chinese centers, we provide the first real-world evidence that radiotherapy, delivered either pre- or postoperatively, is independently associated with improved survival, with 76 and 54% lower risk of mortality, respectively, with the greatest benefit seen in node-negative patients. These observations challenge current guidelines and offer a new clinical perspective.

Biologically, T4 tumors often invade adjacent structures (9), leaving a high likelihood of microscopic residual disease at the circumferential or deep margin. Seto et al. (7) reported a 27% local recurrence rate after R0 resection, predominantly in the original tumor bed and mediastinal nodes, and advocated postoperative irradiation to eradicate occult residuals and reduce locoregional failure. The substantial hazard reduction we observed is consistent with this mechanism: by eliminating microscopic disease and delaying local relapse, postoperative radiotherapy is associated with improved survival in this high-risk population.

The role of postoperative radiotherapy (PORT) in resectable ESCC remains contentious. Single-institution studies by Xiao et al. (21) and Schreiber et al. (22) reported striking 5-year survival advantages (35.1% vs. 13.1% and 28.9% vs. 18.2%, respectively), whereas the randomized trials of Ténière et al. (23) and Fok et al. (24) failed to show any overall benefit. A subsequent systematic review attributed these discrepancies to heterogeneity in tumor stage, nodal status, resection margins and radiotherapy technique, emphasizing the need for precise selection of high-risk patients (25). Current NCCN guidelines therefore recommend active surveillance after R0 resection.

Yet the R0-resected ESCC population is highly heterogeneous, and further substratification may identify subsets that derive meaningful benefit from PORT—specifically T4N0-3M0 tumors. The present study focuses on this subgroup, analyzing treatment modalities and oncological outcomes. PORT was associated with a significant survival advantage across all age groups, indicating that age alone should not be used to deny adjuvant radiotherapy. In the Chinese cohort, age ≥ 60 years and lymphovascular invasion were independent adverse prognostic factors in T4N0-3M0 patients receiving PORT; these variables should be weighed carefully when tailoring adjuvant treatment.

Nodal status also influences the magnitude of benefit. In node-negative patients, PORT produced a markedly greater overall survival (OS) gain. We attribute this “N0 advantage” to a distinct relapse pattern rather than to intrinsic radiosensitivity. Local recurrence dominates in this subset; because systemic dissemination is infrequent when nodes are uninvolved, locoregional relapse becomes the principal barrier to cure. By contrast, node-positive patients already harbor occult systemic micrometastases, and the competing risk of distant failure dilutes any survival gain achievable with local therapy.

The value of postoperative chemotherapy (POCT) is equally controversial. Shen et al. (26) observed significant prolongation of median OS with POCT in node-positive patients, whereas the phase III JCOG 9204 trial (27) found no overall survival benefit. A later systematic review (28) attributed the discordance to heterogeneity in histological subtype, nodal burden and chemotherapy regimens. In our T4N0-3M0 cohort the absence of a significant POCT benefit can be explained by three factors: (1) Biology: T4 tumors are often accompanied by extensive fibrosis and an immunosuppressive microenvironment. After R0 resection, residual disease usually consists of scattered, dormant micrometastases with low proliferative indices, reducing cytotoxic efficacy. (2) Clinical heterogeneity and data limitations: Chemotherapy regimens varied widely in our retrospective cohort—from suboptimal single-agent therapy to modern platinum-taxane doublets—and the SEER database notably lacks specific information on agents, regimens, or cycle numbers, precluding sensitivity analyses stratified by chemotherapy intensity (e.g., platinum-based vs. non-platinum). Even in the Chinese cohort where detailed regimen data were recorded, the small sample size (*n* = 54) and uniform application of postoperative chemotherapy (85.2%) precluded meaningful subgroup comparisons with adequate statistical power. Furthermore, unmeasured confounders such as dose intensity, completion rates, and patient compliance—critical factors influencing chemotherapy efficacy—could not be captured in this retrospective design. This substantial variability introduces statistical noise that may obscure or dilute true therapeutic signals. (3) Statistical power: JCOG 9204 enrolled 242 patients with locally advanced disease; our study included only 95 pN+ and 86 pN0 T4 cases. Taken together, individual chemosensitivity differences, regimen diversity and limited sample size probably account for the observed lack of benefit. Consequently, the null finding regarding POCT may reflect protocol diversity and incomplete data rather than biological ineffectiveness of cytotoxic therapy in this population—a interpretation supported by JCOG 9204, which demonstrated survival benefits only when using standardized cisplatin/5-FU regimens under strict protocol adherence. Nevertheless, both studies suggest that node-positive patients are more likely to gain from chemotherapy. Prospective, adequately powered randomized trials using standard, full-dose regimens specifically in the T4 population are warranted. Yap et al. (29) compared ypT3 and/or ypN+ patients who had received neoadjuvant chemoradiotherapy followed by surgery and found that adjuvant chemoradiotherapy significantly prolonged median OS (31.7 vs. 14.3 months; *p* = 0.004) and median relapse-free survival (18.9 vs. 11.7 months; *p* = 0.020), confirming the independent benefit of adjuvant radiotherapy.

In the present study, univariate analysis of the node-positive subgroup suggested a protective effect of POCT; however, significance was lost after adjustment for age, tumor grade, primary location and other confounders. Several biological mechanisms may explain the absence of chemotherapy benefit in our T4N0-3M0 cohort. First, tumor microenvironment: T4 tumors are characterized by extensive desmoplasia and fibrosis, creating a dense extracellular matrix that impedes drug penetration and delivery to residual tumor cells. Second, dormant micrometastases: Following R0 resection, remaining tumor cells often enter a quiescent, non-proliferative state (G0 phase) with low metabolic activity, rendering them inherently resistant to cytotoxic agents that target actively dividing cells. Third, immunosuppressive milieu: The postoperative microenvironment in T4 disease is enriched with immunosuppressive cytokines and regulatory T-cells, which not only promote tumor survival but may also reduce chemotherapy-induced immunogenic cell death. Fourth, pharmacokinetic challenges: Post-esophagectomy patients frequently experience hemodynamic alterations, delayed gastric emptying (when stomach conduit is used), and nutritional deficiencies, potentially leading to suboptimal drug absorption, increased toxicity, and reduced dose intensity. These factors collectively suggest that cytotoxic therapy may be biologically ineffective against the residual disease burden in fully resected T4 ESCC, contrasting with the locoregional control achieved by radiotherapy. Thus, the biological impact of POCT in T4N0-3M0 ESCC warrants further investigation. Immunotherapy has shown considerable promise in ESCC (30, 31), yet our dataset contains no patients treated with immune agents; this question should be addressed in future trials.

The apparent survival advantage seen in the Chinese cohort relative to SEER should be interpreted cautiously. Specifically, we emphasize that the observed differences are attributable to three key factors: (1) Era effects—87% of Chinese patients were diagnosed in 2010–2021 with modern IMRT, compared with 54.6% of the SEER cohort from 2000 to 2009; (2) Treatment intensity—all Chinese patients received postoperative radiotherapy (vs. only 24.6% in SEER) and 85% received chemotherapy, representing a highly selected, uniform treatment protocol; and (3) Demographic/socioeconomic factors—the Chinese cohort was exclusively Asian and 100% married, whereas SEER included diverse ethnicities with ~50% unmarried/widowed/divorced status, which affects social support and compliance. Thus, the observed ‘superiority’ in outcomes reflects the combined influence of multiple confounders, and it would be inappropriate to conclude that the Chinese cohort inherently outperforms the SEER cohort.

The optimal postoperative radiation field and dose for resected ESCC remains undefined. The contouring protocol currently used by the two Chinese centers (32, 33) defines the clinical target volume (CTV) as the bilateral supraclavicular fossae, upper/middle mediastinal nodal stations and oesophageal tumor bed; for tumors of the lower thoracic oesophagus, the peri-coeliac and adjacent peritoneal nodes are added at the clinician’s discretion. No consensus exists for the dose required after R0 resection of T4 disease; the 50 Gy schedule reported here is provided for reference only and should not be considered definitive.

A pooled analysis of six definitive chemoradiation randomized trials (*n* = 1,722) by Li et al. (34) showed no difference in 3-year overall, progression-free or locoregional relapse-free survival between high-dose (≥59.4 Gy) and standard-dose (50–50.4 Gy) radiotherapy. Similarly, Yao et al. (35) analyzed 199 patients with R0–R1 resected ESCC and found that 50–50.4 Gy produced significantly better 5-year overall and progression-free survival than >50.4 Gy, with lower toxicity; they therefore propose 50–50.4 Gy as the standard postoperative dose. Data specific to pathological (pT4) disease, however, remain scarce.

Our dose–response analysis must be interpreted with substantial caution. Restricted cubic-spline analysis showed no statistically significant association between dose and mortality (P-overall = 0.129; P-non-linear = 0.279). While patients receiving >50 Gy consistently had lower survival probabilities than the 50 Gy reference across all scenarios (HR 1.80, 1.58, and 1.24, respectively), all confidence intervals were wide and included the null value (all *p* > 0.05), indicating no reliable evidence of a dose effect. Given the exploratory nature of these analyses, the small sample size (*n* = 54), and the complete lack of statistical significance, we cannot draw any conclusions about optimal dosing. The 50 Gy schedule should not be interpreted as a recommended or optimal dose; it merely represents the most commonly used dose in this cohort and may serve as a reference point for future hypothesis generation only.

Based on these findings, we suggest that PORT may be preferentially considered for lymph node-negative T4a patients, who showed an 81% lower risk of mortality (HR 0.19); for lymph node-positive patients, given that PORT alone showed only borderline significance (HR 0.36, *p* = 0.058), we recommend combined systemic therapy. Age alone should not be a contraindication; however, careful selection is warranted for patients >80 years with comorbidities. Furthermore, as lymphovascular invasion (LVI) was an independent adverse prognostic factor in the Chinese cohort, LVI-positive patients may require intensified strategies. For future trial design, given the exploratory nature of our dose findings and the lack of statistical significance in all dose comparisons, we suggest that 50–50.4 Gy may serve as a reasonable reference dose based on current practice patterns, with mandatory prospective validation of optimal dosing in adequately powered randomized trials. Finally, given the efficacy of adjuvant nivolumab (CheckMate 577) and our observation of no added benefit from chemotherapy, future trials should evaluate PORT combined with immunotherapy (e.g., PD-1 inhibitors) rather than cytotoxic agents.

This study has several methodological limitations. First, despite propensity-score matching, marked baseline differences persist, deriving primarily from four dimensions of unmeasured confounding: (1) Treatment era and technical platforms: The Chinese cohort (87.0% diagnosed 2010–2021) uniformly received modern IMRT and standardized three-field esophagectomy (100% R0 resection, ≥12 lymph nodes dissected), whereas the SEER cohort (54.6% from 2000 to 2009) encompassed heterogeneous technical evolution from two-dimensional radical radiotherapy to three-dimensional conformal techniques, with only 62.3% of patients having ≥4 lymph nodes removed, reflecting real-world practice variation; (2) Staging accuracy: The Chinese cohort comprised exclusively postoperative pathologically confirmed pT4a (tumor invasion limited to pleura, pericardium or diaphragm), whereas SEER employed mixed clinical (cT4) and pathological (pT4) staging criteria, with potential misclassification bias not addressable by PSM; (3) Tumor burden information: The Chinese cohort had T4a invasion extent confirmed by pathological assessment, whereas SEER lacked quantitative indicators such as tumor size, depth of invasion, SCC-Ag, and CEA; (4) Treatment modality: All patients in the Chinese cohort received postoperative radiotherapy (PORT), whereas SEER included 41.0% preoperative radiotherapy and 34.4% no radiotherapy, with biological differences in treatment sequencing not fully captured. These factors may introduce residual confounding; therefore, cross-cohort comparisons remain exploratory and cannot be interpreted as causal associations. Second, At the two Chinese centers enrolled in this study, the standard-of-care for stage T4 esophageal squamous cell carcinoma is definitive chemoradiotherapy; only a highly selected minority undergo primary surgery. In accordance with institutional protocols, patient preferences, and the surgeons’ intention to maximize locoregional control, all patients with postoperatively confirmed pT4 disease were referred for PORT. Consequently, every patient in the Chinese cohort received PORT, precluding any intra-cohort comparison of locoregional control rates with or without radiotherapy and preventing quantification of the incremental benefit of PORT in this single-arm dataset. Given the rarity of resectable T4 esophageal cancer cases, comprehensive pooled analyses incorporating multi-institutional or multi-regional data remain scarce in the literature. Therefore, we agree that these limitations underscore the urgent need for randomized controlled trials to clarify the role of PORT in this high-risk population. Third, the SEER database lacks key information regarding the basis of T4 staging—whether it represents clinical (cT4) or pT4 classification—and is also missing core treatment parameters such as radiotherapy dose, fractionation, target-volume delineation, and patterns of failure, precluding exclusion of confounding effects from dose–response or target-coverage differences on survival. In contrast, T4 status in the two-center Chinese cohort was strictly defined by postoperative pathology (pT4a), with uniform application of IMRT techniques (clinical target volume routinely encompassing bilateral supraclavicular fossae, upper and middle mediastinal lymph nodes, and tumor bed). Although this staging accuracy and technical homogeneity enhances internal validity, it also indicates that the Chinese cohort represents a highly selected “best-practice” scenario, contrasting with the broad real-world representativeness of SEER, further limiting direct comparability of effect estimates between the two cohorts. Fourth, the SEER registry does not provide radiotherapy dose, fractionation, target-volume delineation, or patterns-of-failure data, precluding a formal dose–response evaluation for the American cohort. This missing information limits our ability to: (1) determine whether the survival benefit observed in SEER is attributable to similar dose ranges as the Chinese cohort (50–50.4 Gy); (2) compare treatment quality between cohorts, given that Chinese patients received standardized IMRT with uniform target volumes (bilateral supraclavicular fossae, mediastinal nodes, and tumor bed), whereas SEER encompassed heterogeneous techniques spanning the 2D, 3D-CRT, and IMRT eras; and (3) assess completion rates or dose intensity, which are critical for interpreting the intention-to-treat effect of radiotherapy. Consequently, the dose–response analysisis restricted to the Chinese cohort and should be considered exploratory rather than definitive. Finally, the retrospective design is inherently susceptible to selection and immortal-time biases, as patients able to tolerate postoperative radiotherapy usually have better baseline performance status. Future validation through prospective randomized controlled trials, propensity-score weighting, or instrumental-variable analyses is warranted.

## 5 Conclusion

This largest-to-date real-world dataset generates the hypothesis that postoperative radiotherapy may be associated with improved survival in selected T4N0-3M0 ESCC patients. These observational findings cannot establish causality. Prospective randomized trials are warranted before clinical adoption.

## Data Availability

The raw data supporting the conclusions of this article will be made available by the authors, without undue reservation.
